# Emerods

**Published:** 1983-04

**Authors:** Dorothy M. H. Tripp

**Affiliations:** Weston-super-Mare


### Letter to the Editor

### "Emerods"

Sir

I should like to congratulate you on the improvement in the Bristol Medico-Chirurgical Journal.

However, I notice on p. 24 of the July/October, 1982, number, in item 12, "Emerods" is taken to mean haemorrhoids. In "The Bible and Modern Medicine", Prof. Rendle Short has a very interesting discussion (chap. 4) on the epidemic in 1 Samuel 5 & 6, which he shows must have been bubonic plague. "The great number of persons infected, the heavy mortality, the spread along the lines of human communication, the tumours or plague boils" (R.V. and margin, for A.V. "emerods"), "and above all the great numbers of dead rodents seen lying about."

"Emerods" does mean haemorrhoids, and the cognate word in Arabic does, too, "but this complaint, common as it is, does not occur in epidemic form, and is seldom fatal. The Hebrew word, 'ophalim', is allied to the name Ophel, which was a mound in Jerusalem. It means a swelling or tumour. . . . The old Greek translation, called the Septuagint, presumably using another Hebrew text, adds "In the midst of the land thereof mice were brought forth, and there was a great and deadly destruction in the city", and later, 'And the land swarmed with mice.'. . . The Hebrews were not exact zoologists, and it is highly probable that the word 'akbar' includes rats as well as mice." The New English Bible has 'rats', 'mice' in a footnote; they are 'rats' in the Jerusalem Bible. In both versions the Philistines had to make models in gold of their 'tumours' and the 'rats', though in the N.E.B. later, chap. 6, verses 11, 17, the tumours are called 'haemorrhoids'.

Yours sincerely,

